# Supplementary material for: Pathophysiological Significance of Neutrophilic Transfer RNA-Derived Small RNAs in Asymptomatic Moyamoya Disease
Source: Cells. 2021 May 1;10(5):1086. doi: 10.3390/cells10051086 (PMC8147334; doi:10.3390/cells10051086)

2D Structure	Local AU	Position

```
213                              8mer-1a   243
 5'-gaGAAACCACGTGAATGTAATGA--ATGTGGGa-3' utr
       ||| ||    |:|  :|:  | ||  |||||||
 3'-ucCUUAGG---AUUGGCGAU-CUGGUACACCCu-5' tRF
                   16151413      7 6 5 4 3 2
                  3' Pairing       Seed
```

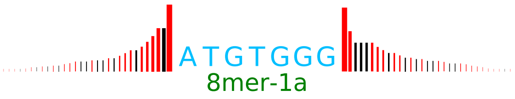

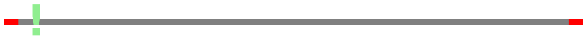

2D Structure	Local AU	Position

```
72                              8mer-1a   101
 5'-agaGATCCTAAAGGTGTAATGTATGTGGGa-3' utr
         :||||||||  |:  ||    :|||||||
 3'-uccUUAGGAUUGGCG-AUCUGGUACACCCu-5' tRF
                 1615 1413      7 6 5 4 3 2
                 3' Pairing       Seed
```

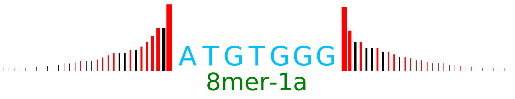

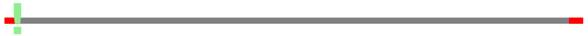

Supplement: Supplementary file 1 [file cells-10-01086-s001.zip › cells-1149161 3rd supplementary materials/Data Supplements/Data Supplement V.zip!/Data supplement 6-mRNAs targets of tsRNAs/tsRNA-Glu-TTC-010/Diagrams/tRF-Glu-TTC-010_vs_ENST00000295208(ZNF514).pdf]
